# Association between triglyceride levels and cardiovascular disease in patients with acute pancreatitis

**DOI:** 10.1371/journal.pone.0179998

**Published:** 2018-01-30

**Authors:** Laurel A. Copeland, C. Scott Swendsen, Dawn M. Sears, Andrea A. MacCarthy, Catherine J. McNeal

**Affiliations:** 1 VA Central Western Massachusetts Healthcare System, Leeds, Massachusetts, United States of America; 2 Department of Medicine, Baylor Scott & White Health, Temple, Texas, United States of America; 3 UT Health San Antonio, San Antonio, Texas, United States of America; 4 Texas A&M Health Science Center, Temple, Texas, United States of America; 5 Department of Internal Medicine, Gastroenterology Service, Baylor Scott & White Health, Temple, Texas, United States of America; 6 Center of Excellence for Research on Returning War Veterans, Central Texas Veterans Health Care System, Waco, Texas, United States of America; University of Szeged, HUNGARY

## Abstract

Conventional wisdom supports prescribing “fibrates before statins”, that is, prioritizing treatment of hypertriglyceridemia (hTG) to prevent pancreatitis ahead of low-density lipoprotein cholesterol to prevent coronary heart disease. The relationship between hTG and acute pancreatitis, however, may not support this approach to clinical management. This study analyzed administrative data from the Veterans Health Administration for evidence of (1) temporal association between assessed triglycerides level and days to acute pancreatitis admission; (2) association between hTG and outcomes in the year after hospitalization for acute pancreatitis; (3) relative rates of prescription of fibrates vs statins in patients with acute pancreatitis; (4) association of prescription of fibrates alone versus fibrates with statins or statins alone with rates of adverse outcomes after hospitalization for acute pancreatitis. Only modest association was found between above-normal or extremely high triglycerides and time until acute pancreatitis. CHD/MI/stroke occurred in 23% in the year following AP, supporting cardiovascular risk management. Fibrates were prescribed less often than statins, defying conventional wisdom, but the high rates of cardiovascular events in the year following AP support a clinical focus on reducing cardiovascular risk factors.

## Introduction

Lederle et al. noted that the risk of acute pancreatitis (AP) attributable to hypertriglyceridemia (hTG) and the extent to which hTG is an epiphenomenon rather than a cause of AP, raised critical questions that have not been answered despite longstanding guidelines emphasizing the need to treat hTG to prevent AP [1]. In addition, research suggests that statins and not fibrates may better reduce the risk of AP in patients with hTG due to favorable changes in cholesterol-associated gallstone formation [2]. Alcohol abuse and gallstone disease account for approximately 80% of AP, with hTG estimated to contribute 3–10% [3]. Despite the low risk of hTG-induced pancreatitis [4], lipid guidelines for primary and secondary prevention of cardiovascular disease (CVD) recommend as a first step, triglyceride-lowering therapy (i.e., fibrate) rather than statin therapy in patients with hTG >500 mg/dL to prevent hTG-associated pancreatitis[5] although greater risk of pancreatitis may be associated with levels well above 2000 mg/dL [6,7].

To understand the relationships among hTG, AP, lipid-lowering drugs (LLRx), and CVD outcomes among patients with acute pancreatitis, we analyzed data from a large, integrated health care system, the Veterans Health Administration (VA), to determine the following: (1) the temporal association between days to AP admission and TG level; (2) associations between high TG and adverse outcomes after AP admission (1-year mortality; gall bladder disease; stroke; coronary heart disease (CHD); coronary artery bypass graft (CABG); and percutaneous intervention (PCI); (3) relative rates of prescription of fibrates vs statins in patients with AP; (4) association of prescription of fibrates alone rather than fibrates with statins or statins alone with rates of adverse outcomes following AP. We expected a negative correlation between lag to AP admission and TG level and anticipated positive associations between adverse outcomes and both very high TG and use of fibrates alone.

## Materials and methods

### Data source and sample

The Institutional Review Boards of the Central Texas Veterans Health Care System and of the VA Central Western Massachusetts Healthcare System approved the study using an expedited review and waiver of informed consent. The Scott & White Memorial Hospital Institutional Review Board exempted the study from oversight at Baylor Scott & White. Data were extracted from the STOPP data repository, a collection of administrative extracts from the VA’s all-electronic medical records system (EMR) on all patients treated during fiscal years 2006–2009 (FY2006-FY2009; Oct 2005-Sep 2009) [8] and from the VA’s Corporate Data Warehouse. Variables included dates and types of care, diagnoses, procedures, prescription medications, lab test results, outpatient care, and admission and inpatient stay details on 7 million veterans of US military service using the Veterans Health Administration for care. Administrative data are reported nightly from all facility EMR’s across the VA system and cumulated in the Corporate Data Warehouse and related files, accessible for research purposes via a secure VA intranet environment after IRB approval.

Inclusion criteria were age 18 years or older, veterans granted VA Priority 1–8 indicating eligibility for VA care based on military service or disability or poverty, valid triglycerides lab data, and admitted for AP during FY2006-FY2009. For patients with multiple qualifying admissions, their first during the observation period was used.

### Measures

Variables assessed age, race, ethnicity, gender, marital status, and utilization (lab results; hospital admission; timing of care) as well as risk adjustment measures including mental and physical illnesses and the Charlson Comorbidity Index of conditions associated with post-discharge one-year mortality calculated without the two cancer measures (retained as separate indicators) [9,10].

The inclusion criterion for AP was defined by International Classification of Diseases 9th Edition (ICD9) diagnosis code 577.0 during an inpatient admission; chronic pancreatitis was defined by ICD9 code 577.1 (inpatient or outpatient diagnosis). The date of first AP admission during the observation period was the index date for each patient. Covariates included TG level, the maximum recorded lab result on the date closest to AP admission in the year prior to AP admission through the first week prior to admission. Lag between TG values and AP admission was determined in days, also categorized into quarters (9 to 12 months prior, 6 to 9 months prior, 3 to 6 months prior, and 0 to 3 months prior through 7 days pre-admission). TG values from -7 through +7 days were captured to examine hTG at the time of hospitalization for AP, in “month 0”. Based on American Heart Association guidelines published by Miller et al. 2011, TG values <150 mg/dL are categorized as normal, 150–199 mg/dL are borderline, 200–500 are very high [11]. However, the lack of evidence supporting the definition of 500+ as critically high led us to recategorize TG values for analysis as follows: <200 (normal/borderline), 200–499, 500–999, 1000–1999, 2000+; dichotomies of below and above 500, 1000, and 2000 were also created.

To ascertain and adjust for baseline comorbidity, the Charlson Comorbidity Index and other relevant disorders were assessed from International Classification of Diseases 9^th^ Edition (ICD-9) diagnoses recorded in the 365 days prior to AP admission. Conditions and the corresponding ICD-9 codes included the Charlson conditions of myocardial infarction (AMI; 410, 412), chronic heart failure (428), peripheral vascular disease, stroke (430–438), dementia (290), chronic obstructive pulmonary disease (490–496, 501–506.4), rheumatologic disease (710.0, 710.1, 710.4, 714.0, 714.1, 714.2, 714.81, 725), peptic ulcer (531–534), cirrhosis of the liver (571.2, 571.4, 571.5, 571.6), liver failure (572.2–572.8, 456.0, 456.1, 456.20, 456.21), diabetes (250), hemi/paraplegia (342, 344.1), chronic kidney disease (582–583, 585–586, 588), cancers (140–172, 174–195, 200–208), metastatic cancers (196, 197, 198, 199), AIDS (042–044); as well as CHD (414), gall bladder disease (574, 575, 576), osteoporosis (733), hypertension (401–405), hypothyroidism (244), and vitamin D deficiency (268). Additional conditions were assessed to understand variability in risk. Previous research has noted differences in post-hospitalization health outcomes for veterans with mental and behavioral disorders [8]. Alcohol disorders were indicated by ICD-9 diagnoses of 291, 303, or 305.0. Serious mental illnesses as defined by the VA [12] were major depressive disorder (MDD; 296.2, 296.3, 311), posttraumatic stress disorder (PTSD; 309.81), and psychotic disorders (bipolar disorder, 296.0–296.1, 296.4–296.8; and schizophrenia, 295). Prior-year AP admission during the 365 days before the index admission was identified from inpatient data. Prescription drug use was noted for statins, fibrates, and other lipid-lowering medications (LLRx) prescribed for at least 60 days in the 45–75 days prior to admission (see S1 Appendix).

### Dependent variables

Adverse outcomes of acute pancreatitis include recurrent gall bladder disease and multiple organ failure and death; risks from uncontrolled lipids (primarily LDL-cholesterol) include coronary heart disease, stroke, and myocardial infarct. Outcomes in the 365 days after the index AP admission date therefore included gallbladder disease, cardiovascular disease (CVD) events and related procedures (CHD, stroke, MI, PCI, CABG), and death within 1 year post-AP (see S1 Appendix).

### Analysis

To address the first aim, maximum TG level was graphed by month prior to AP admission to show temporal relationship of elevated TG with acute AP exacerbation. A Cox proportional hazards model estimated the association between TG level and time from TG assessment to AP admission adjusting for demographic and clinical covariates. To address the third aim regarding treatment with fibrates or statins, chi-square analysis assessed frequencies of and bivariate associations between receipt of fibrate, statins, or both and TG level. To address the second and fourth aims regarding adverse outcomes associated with LLRx patterns and outcomes following AP hospitalization, multivariable logistic regression modeled the association of LLRx treatment (fibrate-alone, statin+fibrate, statins-alone, neither fibrate nor statins and other LLRx (e.g., ezetimibe, niacin) and TG level with post-AP adverse events (gall bladder disorders, CHD, stroke, MI, PCI but not CABG as it was too rare to model), any of those clinical outcomes, one-year mortality, and the combined outcome (any adverse clinical outcome or death). The models were repeated on the subsample without alcohol or gall bladder disorders diagnosed pre- or post-AP.

## Results

The cohort of 20,608 veterans with AP during FY2006-FY2009 included 19,778 males (96%), age 21–97 (mean: 60.4, SD: 12.2), of whom 27% were black, 3% Asian/other; 7% were Hispanic; 40% were married (see Table 1). About one-third had diagnosis of either alcohol or gall bladder disorders (6,336; 31%). Comorbidity levels in the baseline pre-AP year averaged 2.0 (SD 2.1) on the adapted Charlson index of mortal conditions (omitting cancer measures); the most common physical disorders were hypertension (77%), dyslipidemia (56%), and diabetes (40%). Gall bladder disorders characterized 6%; 13.1% had cancer and 2.6% had metastatic solid tumor (13.4% had either or both). Regarding behavioral and mental disorders, about one-quarter (25%) had an alcohol use disorder, 33% were depressed, 15% had PTSD, and 10% had a psychotic disorder. In the year following AP admission, 36% of patients had an adverse clinical outcome (MI, CHD, stroke, CABG, PCI), and 13% of patients died including 14% of those without alcohol or gall bladder disorders.

**Table 1:** Characteristics of VA patients admitted with acute pancreatitis FY2006-FY2009 (n = 20,608).

Characteristic	Mean	Standard Deviation
Age in years	60.3	12.2
Charlson Comorbidity Index minus cancers	2.0	2.1
Triglycerides level pre-admission	237.4	562.6
Lag in days from triglyceride assay to admission	135.5	95.5
LDL-Cholesterol level pre-admission (n = 13,785; 31% no LDL-C labs)	98.2	37.6
HDL-Cholesterol level pre-admission (n = 14,712; 26% no HDL-C labs)	44.0	19.0
	N	Percentage
Female	830	4.0
Race (557 cases missing data)		
White	14,118	70.4
African American	5,389	26.9
Asian, Native American, other races	544	2.7
Hispanic (557 cases missing data)	1,381	6.9
Married (31 cases missing data)	8,277	40.2
Hypertension	15,916	77.2
Dyslipidemia	11,472	55.6
Hypertriglyceridemia—index admission	657	3.2
Acute pancreatitis, prior year	215	1.0
Cancer, prior year	2,695	13.1
Metastatic cancer, prior year	536	2.6
Alcohol disorders, prior year	5,244	25.4
Posttraumatic stress disorder, prior year	3,193	15.5
Major depressive disorder, prior year	6,736	32.7
Bipolar disorder or Schizophrenia, prior year	2,145	10.4
Triglycerides category, nearest pre-admission		
<200 mg/dL	14,686	71.3
200–499 mg/dL	4,669	22.7
500–999 mg/dL	731	3.6
1000–1999 mg/dL	286	1.4
2000 mg/dL or higher	236	1.2
Statins within 2 months pre-admission	5,017	24.3
Fibrates within 2 months pre-admission	1,112	5.4
Statins and fibrates within 2 months pre-admission	498	2.4
Statins only within 2 months pre-admission	4,519	21.9
Fibrates only within 2 months pre-admission	614	3.0
LLRx other within 2 months pre-admission	645	3.1
Died within 1 year of AP admission	2,750	13.3
CHD within 1 year of AP admission	3,461	16.8
Stroke within 1 year of AP admission	1,560	7.6
MI within 1 year of AP admission	1,089	5.3
PCI within 1 year of AP admission	546	2.7
CABG within 1 year of AP admission	73	0.35
CHD, Stroke, or MI within 1 year of AP admission	4,641	22.5
CHD, Stroke, MI, PCI, CABG within 1 year of AP admission (combined clinical outcome)	7,519	36.5
Any clinical outcome including death within 1 year of AP admission (combined outcome)	9,407	45.7

### Bivariate associations

#### Association between hypertriglyceridemia and adverse outcomes

Increasing TG level in the year prior to AP admission was associated with decreasing 1-year mortality in unadjusted analysis (Mantel-Haenszel chi-square test-for-trend 119.5, df = 1, p < .0001) with 15% of patients in the lowest category (TG<200) dying within one year, 8% with TG 200–499 mg/dL and decreasing percentages to 4% for those in the highest category (TG 2000 or higher). Age correlated slightly negatively with TG level meaning younger patients had relatively higher TG (r = -0.14; p < .0001).

#### Association between TG level and days until admission

**Fig 1:**
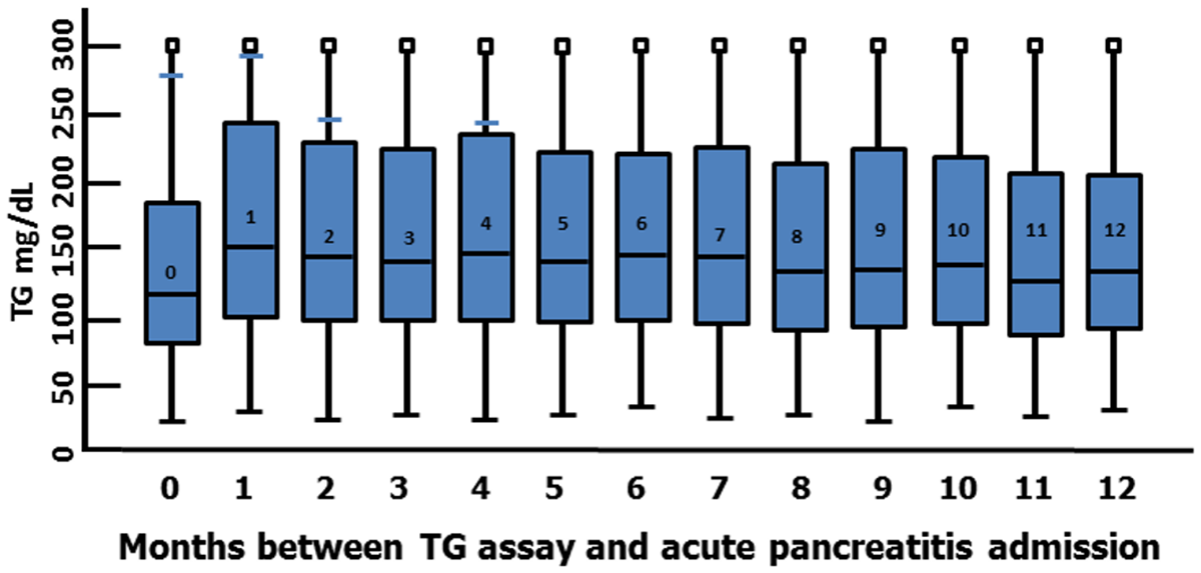
Mean triglyceride level by months until admission for acute pancreatitis among 20,608 veterans (FY2006-FY2009).

The number of days to AP admission from TG assessment varied significantly by TG level with shorter lag to admission among those with very high TG, ranging from 108.5 days for TG<200 to 90.9 days for TG 1000–1999 and 54.5 days for TG of 2000+ (F = 23.8; df = 4, 20603; p < .0001). Overall, TG level correlated modestly and negatively with days until admission for acute pancreatitis (r = -.07; p < .0001; see Fig 1), consistent with a temporal relationship between TG level and risk of AP assuming TG levels continued to increase between last lab test and AP admission. The adjusted Cox model estimated a small positive association between days from TG assay to AP admission and hTG (TG of 2000 mg/dL or more; HR = 1.38 95%CI 1.16–1.63) but not with lesser levels of elevated TG, in the range 200–1999 mg/dL.

#### Relative rates of prescription of fibrates vs statins

Most patients with AP were not taking LLRx prior to their admission for AP: 24% were on statins, 5% fibrates including 2% on both drugs, and 3% were on other LLRx. Thus, fibrates were less commonly prescribed than statins in patients hospitalized for AP. Patients with above-borderline/normal TG levels were more likely to be on fibrates only (65% of 614) or both fibrates and statins (61% of 498) than on neither (26% of 14,977) or statins only (30% of 4,519; chi-square 724.4; p < .0001). However, only 5% of all AP patients were in the fibrates-treated groups.

### Multivariable models

#### Association of prescription pattern with adverse outcomes

Controlling for demographics, prior-year acute pancreatitis, lag between TG assay and AP admission, Charlson comorbidity and cancers, alcohol and psychiatric disorders, hypertension, dyslipidemia, and prescribed LLRx, elevated triglyceride levels—as opposed to normal/borderline levels—were significantly inversely related to post-AP gall bladder disease, one-year mortality, CHD, and the combined clinical outcome as well as the combined clinical and mortality outcome; odds ratios ranged from 0.29 to 0.91 (see Table 2). Statins and fibrates were inversely associated with 1-year mortality, but were risk factors in the models of other clinical outcomes. Triglyceride level was not significantly related to the post-AP outcomes of stroke, MI, or PCI.

**Table 2:** Factors associated with post-acute pancreatitis outcomes among 20,608 VA patients (FY2006-FY2009).

**Outcome = CHD**				
TG 2000+ vs <200 mg/dL	0.54	0.32	0.88	*
TG 1000–1999 vs <200 mg/dL	0.65	0.43	0.99	*
TG 500–999 vs <200 mg/dL	1.09	0.88	1.35	
TG 200–499 vs <200 mg/dL	1.07	0.98	1.18	
Lag between TG assay and AP admission	1.00	1.00	1.00	
Age in decades	1.48	1.43	1.54	*
Female	0.61	0.46	0.81	*
African American	0.64	0.58	0.72	*
Asian or other race	0.81	0.63	1.05	
Hispanic	0.71	0.61	0.84	*
Married	1.02	0.94	1.11	
Acute pancreatitis in prior year	^a^			
Charlson comorbidity score (excluding cancers)	1.22	1.20	1.24	*
Cancers, non-metastatic	0.92	0.81	1.04	
Cancer, metastasized tumor	0.75	0.57	0.99	*
Alcohol disorder	0.84	0.75	0.95	*
Dyslipidemia	2.30	2.08	2.54	*
Hypertension	1.70	1.49	1.93	*
Depression	1.13	1.03	1.24	*
Post-traumatic stress disorder	0.95	0.84	1.07	
Bipolar disorder or schizophrenia	0.88	0.75	1.03	
Statins	1.74	1.59	1.89	*
Fibrates	0.93	0.79	1.09	
Other LLRx	1.76	1.47	2.11	*
**Outcome = Stroke**				
TG 2000+ vs <200 mg/dL	0.95	0.49	1.82	
TG 1000–1999 vs <200 mg/dL	0.62	0.32	1.24	
TG 500–999 vs <200 mg/dL	0.97	0.70	1.33	
TG 200–499 vs <200 mg/dL	1.03	0.91	1.18	
Lag between TG assay and AP admission	1.00	1.00	1.00	
Age in decades	1.50	1.42	1.57	*
Female	0.88	0.62	1.26	
African American	0.94	0.83	1.08	
Asian or other race	1.14	0.83	1.57	
Hispanic	0.85	0.69	1.05	
Married	1.08	0.96	1.20	
Acute pancreatitis in prior year	^a^			
Charlson comorbidity score (excluding cancers)	1.25	1.22	1.27	*
Cancers, non-metastatic	0.99	0.85	1.16	
Cancer, metastasized tumor	0.52	0.34	0.80	*
Alcohol disorder	1.02	0.88	1.19	
Dyslipidemia	1.14	1.01	1.29	*
Hypertension	1.54	1.30	1.82	*
Depression	1.07	0.94	1.21	
Post-traumatic stress disorder	1.05	0.90	1.24	
Bipolar disorder or schizophrenia	1.03	0.85	1.26	
Statins	1.31	1.16	1.48	*
Fibrates	0.81	0.64	1.03	
Other LLRx	1.16	0.89	1.51	
**Outcome = MI**				
TG 2000+ vs <200 mg/dL	0.62	0.27	1.43	
TG 1000–1999 vs <200 mg/dL	0.56	0.26	1.22	
TG 500–999 vs <200 mg/dL	1.18	0.84	1.65	
TG 200–499 vs <200 mg/dL	1.15	0.99	1.33	
Lag between TG assay and AP admission	1.00	1.00	1.00	
Age in decades	1.29	1.22	1.37	*
Female	0.93	0.62	1.40	
African American	0.76	0.65	0.90	*
Asian or other race	0.65	0.41	1.03	
Hispanic	0.94	0.73	1.20	
Married	0.95	0.84	1.09	
Acute pancreatitis in prior year	^a^			
Charlson comorbidity score (excluding cancers)	1.28	1.25	1.31	*
Cancers, non-metastatic	0.95	0.79	1.14	
Cancer, metastasized tumor	0.70	0.44	1.13	
Alcohol disorder	0.85	0.71	1.02	
Dyslipidemia	1.70	1.45	1.99	*
Hypertension	1.14	0.94	1.38	
Depression	1.04	0.90	1.21	
Post-traumatic stress disorder	0.80	0.65	0.98	*
Bipolar disorder or schizophrenia	0.81	0.63	1.04	
Statins	1.39	1.21	1.60	*
Fibrates	0.86	0.66	1.11	
Other LLRx	1.55	1.19	2.01	*
**Outcome = CHD/Stroke/MI**				
TG 2000+ vs <200 mg/dL	0.58	0.37	0.90	*
TG 1000–1999 vs <200 mg/dL	0.64	0.44	0.94	*
TG 500–999 vs <200 mg/dL	1.07	0.88	1.31	
TG 200–499 vs <200 mg/dL	1.05	0.96	1.14	
Lag between TG assay and AP admission	1.00	1.00	1.00	
Age in decades	1.54	1.48	1.59	*
Female	0.70	0.56	0.89	*
African American	0.75	0.69	0.82	*
Asian or other race	0.81	0.65	1.03	
Hispanic	0.74	0.64	0.86	*
Married	1.02	0.95	1.10	
Acute pancreatitis in prior year	^a^			
Charlson comorbidity score (excluding cancers)	1.25	1.23	1.27	*
Cancers, non-metastatic	0.97	0.87	1.08	
Cancer, metastasized tumor	0.66	0.51	0.85	*
Alcohol disorder	0.91	0.82	1.01	
Dyslipidemia	1.83	1.68	1.99	*
Hypertension	1.62	1.45	1.80	*
Depression	1.10	1.01	1.20	*
Post-traumatic stress disorder	0.97	0.87	1.08	
Bipolar disorder or schizophrenia	0.92	0.81	1.06	
Statins	1.65	1.52	1.78	*
Fibrates	0.87	0.75	1.01	
Other LLRx	1.63	1.37	1.95	*
**Outcome = PCI**				
TG 2000+ vs <200 mg/dL	0.44	0.14	1.39	
TG 1000–1999 vs <200 mg/dL	0.71	0.31	1.62	
TG 500–999 vs <200 mg/dL	1.02	0.65	1.60	
TG 200–499 vs <200 mg/dL	1.00	0.82	1.24	
Lag between TG assay and AP admission	1.00	1.00	1.00	
Age in decades	1.03	0.95	1.12	
Female	1.18	0.74	1.87	
African American	0.96	0.78	1.18	
Asian or other race	1.16	0.71	1.91	
Hispanic	0.90	0.64	1.28	
Married	0.88	0.73	1.05	
Acute pancreatitis in prior year	2.13	1.03	4.43	*
Charlson comorbidity score (excluding cancers)	1.21	1.17	1.25	*
Cancers, non-metastatic	1.03	0.79	1.33	
Cancer, metastasized tumor	0.55	0.26	1.14	
Alcohol disorder	0.76	0.60	0.96	*
Dyslipidemia	1.34	1.09	1.64	*
Hypertension	1.61	1.23	2.12	*
Depression	1.00	0.82	1.22	
Post-traumatic stress disorder	1.23	0.97	1.56	
Bipolar disorder or schizophrenia	0.91	0.67	1.24	
Statins	1.26	1.04	1.54	*
Fibrates	1.23	0.89	1.71	
Other LLRx	1.28	0.87	1.89	
**Outcome = Gall bladder disease**				
TG 2000+ vs <200 mg/dL	0.29	0.17	0.51	*
TG 1000–1999 vs <200 mg/dL	0.36	0.23	0.57	*
TG 500–999 vs <200 mg/dL	0.56	0.44	0.71	*
TG 200–499 vs <200 mg/dL	0.91	0.84	1.00	
Lag between TG assay and AP admission	1.00	1.00	1.00	
Age in decades	1.30	1.26	1.35	*
Female	1.67	1.40	1.99	*
African American	0.62	0.56	0.68	*
Asian or other race	0.86	0.68	1.08	
Hispanic	1.22	1.07	1.40	*
Married	1.15	1.07	1.24	*
Acute pancreatitis in prior year	^a^			
Charlson comorbidity score (excluding cancers)	0.96	0.94	0.98	*
Cancers, non-metastatic	0.81	0.73	0.91	*
Cancer, metastasized tumor	0.46	0.35	0.62	*
Alcohol disorder	0.49	0.44	0.55	*
Dyslipidemia	1.21	1.12	1.32	*
Hypertension	0.94	0.85	1.03	
Depression	0.86	0.79	0.94	*
Post-traumatic stress disorder	1.00	0.89	1.12	
Bipolar disorder or schizophrenia	0.77	0.66	0.89	*
Statins	0.96	0.88	1.05	
Fibrates	1.08	0.92	1.27	
Other LLRx	1.00	0.82	1.22	
**Outcome = any clinical event**				
TG 2000+ vs <200 mg/dL	0.42	0.29	0.61	*
TG 1000–1999 vs <200 mg/dL	0.43	0.31	0.59	*
TG 500–999 vs <200 mg/dL	0.75	0.63	0.89	*
TG 200–499 vs <200 mg/dL	0.95	0.88	1.03	
Lag between TG assay and AP admission	1.00	1.00	1.00	
Age in decades	1.46	1.42	1.50	*
Female	1.42	1.20	1.67	*
African American	0.67	0.62	0.72	*
Asian or other race	0.81	0.66	0.98	*
Hispanic	1.00	0.88	1.13	
Married	1.09	1.02	1.16	*
Acute pancreatitis in prior year	0.14	0.07	0.29	*
Charlson comorbidity score (excluding cancers)	1.14	1.12	1.15	*
Cancers, non-metastatic	0.89	0.80	0.98	*
Cancer, metastasized tumor	0.51	0.41	0.63	*
Alcohol disorder	0.65	0.60	0.70	*
Dyslipidemia	1.47	1.37	1.58	*
Hypertension	1.18	1.09	1.28	*
Depression	0.96	0.90	1.04	
Post-traumatic stress disorder	1.00	0.91	1.09	
Bipolar disorder or schizophrenia	0.86	0.77	0.96	*
Statins	1.35	1.25	1.46	*
Fibrates	0.93	0.81	1.07	
Other LLRx	1.34	1.14	1.59	*
**Died within 1 year post-AP**				
TG 2000+ vs <200 mg/dL	0.64	0.33	1.22	
TG 1000–1999 vs <200 mg/dL	0.53	0.28	0.98	*
TG 500–999 vs <200 mg/dL	0.66	0.49	0.90	*
TG 200–499 vs <200 mg/dL	0.86	0.77	0.96	*
Lag between TG assay and AP admission	1.00	1.00	1.00	
Age in decades	1.48	1.42	1.54	*
Female	0.68	0.50	0.92	*
African American	0.81	0.73	0.90	*
Asian or other race	0.70	0.52	0.94	*
Hispanic	0.95	0.81	1.13	
Married	0.98	0.89	1.07	
Acute pancreatitis in prior year	0.90	0.56	1.46	
Charlson comorbidity score (excluding cancers)	1.23	1.21	1.25	*
Cancers, non-metastatic	1.83	1.63	2.05	*
Cancer, metastasized tumor	6.70	5.47	8.21	*
Alcohol disorder	1.22	1.09	1.37	*
Dyslipidemia	0.68	0.61	0.75	*
Hypertension	0.79	0.71	0.88	*
Depression	0.87	0.78	0.96	*
Post-traumatic stress disorder	0.88	0.77	1.00	
Bipolar disorder or schizophrenia	0.92	0.78	1.09	
Statins	0.90	0.80	1.00	
Fibrates	0.92	0.74	1.14	
Other LLRx	1.04	0.81	1.34	
**Combined clinical outcome or death**				
TG 2000+ vs <200 mg/dL	0.45	0.32	0.63	*
TG 1000–1999 vs <200 mg/dL	0.43	0.32	0.58	*
TG 500–999 vs <200 mg/dL	0.69	0.58	0.82	*
TG 200–499 vs <200 mg/dL	0.91	0.84	0.98	*
Lag between TG assay and AP admission	1.00	1.00	1.00	
Age in decades	1.60	1.55	1.65	*
Female	1.31	1.11	1.53	*
African American	0.65	0.61	0.70	*
Asian or other race	0.71	0.59	0.86	*
Hispanic	1.03	0.91	1.16	
Married	1.06	1.00	1.13	
Acute pancreatitis in prior year	0.34	0.23	0.52	*
Charlson comorbidity score (excluding cancers)	1.20	1.19	1.22	*
Cancers, non-metastatic	1.19	1.08	1.32	*
Cancer, metastasized tumor	2.54	2.05	3.15	*
Alcohol disorder	0.74	0.68	0.80	*
Dyslipidemia	1.19	1.11	1.28	*
Hypertension	1.03	0.95	1.11	
Depression	0.90	0.84	0.96	*
Post-traumatic stress disorder	1.00	0.92	1.09	
Bipolar disorder or schizophrenia	0.86	0.78	0.96	*
Statins	1.26	1.17	1.36	*
Fibrates	0.94	0.82	1.08	
Other LLRx	1.30	1.09	1.55	*

^a^ inestimable

* indicates 95% CI omits 1.00 (statistically significant effect)

Among those without alcohol or gall bladder disorders, risk of 1-year mortality was lower for those with elevated TG (results not shown). The models of MI, CHD, and stroke found no association with TG level. The combined cardiovascular outcome showed decreased risk associated with fibrates and increased risk associated with statins and other LLRx, at modest levels.

## Discussion

Our examination of patient data from the year prior to admission with AP through the year following admission found little supportive evidence that “fibrates before statins” in patients with elevated TG, 200 mg/dL or higher, was a meaningful strategy. The patients studied were veterans of military service, mostly males and highly comorbid, therefore the results observed may not apply to women or patients in private healthcare systems. Patients may have had lipid assays or care in other healthcare systems leading to underreporting. Increasing TG level did appear to be weakly associated with admission for AP but generally speaking not with subsequent adverse outcomes. Rather, the inverse association between elevated TG and post-AP death or cardiovascular outcomes suggests that addressing risk factors for cardiovascular outcomes would be a better focus of care than lowering TG. The use of statins, fibrates, and other lipid-lowering drugs did not appear to follow the expected pattern–“fibrates before statins”—and appeared to serve mainly to identify patients at elevated risk of MI, CHD, stroke, PCI, or CABG.

## Supporting information

S1 Appendix Variable definitions. (TIF)
