# Supplementary figures and images for: Association between triglyceride levels and cardiovascular disease in patients with acute pancreatitis
Source: PLoS One. 2018 Jan 30;13(1):e0179998. doi: 10.1371/journal.pone.0179998 (PMC5790224; doi:10.1371/journal.pone.0179998)

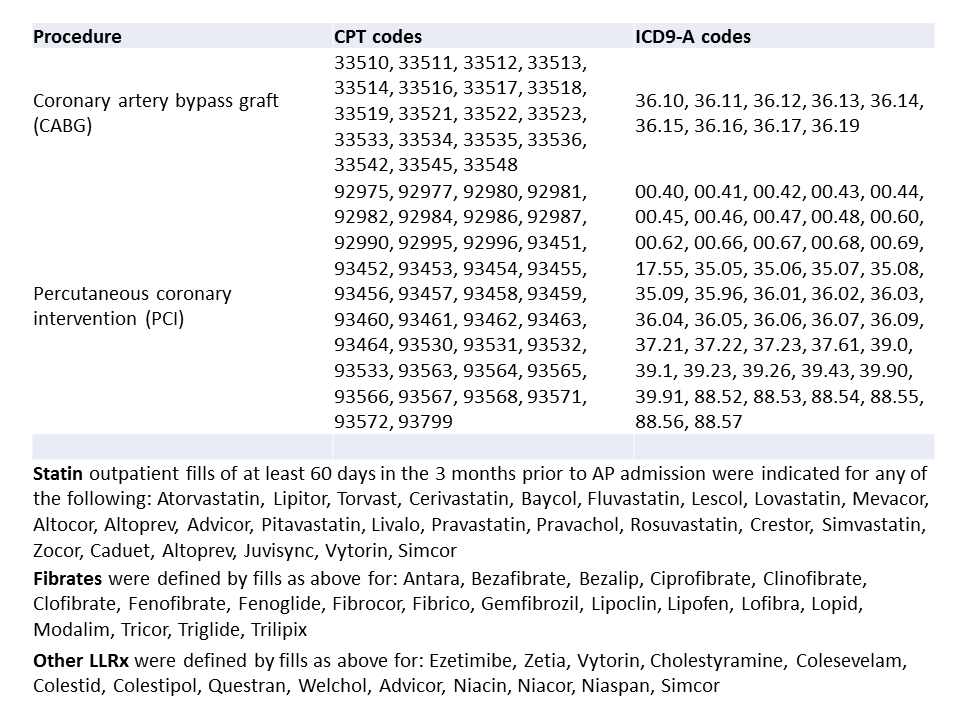

Supplement: S1 Appendix — (TIF) [file pone.0179998.s001.tif]
